# Disturbed Cyclical Stretch of Endothelial Cells Promotes Nuclear Expression of the Pro-Atherogenic Transcription Factor NF-κB

**DOI:** 10.1007/s10439-016-1750-z

**Published:** 2016-10-27

**Authors:** Ryan M. Pedrigi, Konstantinos I. Papadimitriou, Avinash Kondiboyina, Sukhjinder Sidhu, James Chau, Miten B. Patel, Daniel C. Baeriswyl, Emmanuel M. Drakakis, Rob Krams

**Affiliations:** Department of Bioengineering, Imperial College London, Prince Consort Rd., London, SW7 2AZ UK

**Keywords:** Atherosclerosis, Mechanobiology, Biomechanics, Strain, Shear stress, Fluid–structure interaction, Advanced plaques, Thin cap fibroatheroma, Nuclear factor kappa b

## Abstract

Exposure of endothelial cells to low and multidirectional blood flow is known to promote a pro-atherogenic phenotype. The mechanics of the vessel wall is another important mechano-stimulus within the endothelial cell environment, but no study has examined whether changes in the magnitude and direction of cell stretch can be pro-atherogenic. Herein, we developed a custom cell stretching device to replicate the *in vivo* stretch environment of the endothelial cell and examined whether low and multidirectional stretch promote nuclear translocation of NF-κB. A fluid–structure interaction model of the device demonstrated a nearly uniform strain within the region of cell attachment and a negligible magnitude of shear stress due to cyclical stretching of the cells in media. Compared to normal cyclical stretch, a low magnitude of cyclical stretch or no stretch caused increased expression of nuclear NF-κB (*p* = 0.09 and *p* < 0.001, respectively). Multidirectional stretch also promoted significant nuclear NF-κB expression, comparable to the no stretch condition, which was statistically higher than the low (*p* < 0.001) and normal (*p* < 0.001) stretch conditions. This is the first study to show that stretch conditions analogous to atherogenic blood flow profiles can similarly promote a pro-atherogenic endothelial cell phenotype, which supports a role for disturbed vessel wall mechanics as a pathological cell stimulus in the development of advanced atherosclerotic plaques.

## Introduction

Coronary artery disease is the worldwide leading cause of death.^27^ It is characterized as a chronic lipid-driven inflammatory disease that manifests as atherosclerotic plaques composed of a lipid-rich necrotic core and immune cells within the intima of the coronary arteries.^11^,^27^ Plaque development requires a dysfunctional endothelium (the normal endothelium regulates the passage of proteins and cells from the bloodstream into the vessel wall) that results in expression of pro-inflammatory mediators such as NF-κB, disruption of cell–cell junctions, and expression of leukocyte adhesion molecules (e.g., vascular cell adhesion molecule-1). The precise environmental cues that promote a dysfunctional endothelium are not well characterized, but the tissue mechanical environment is known to play an important role.^11^,^26^

Endothelial cells are highly mechano-sensitive^9^,^11^ and most investigators have focused on their response to fluid shear stress, which is a blood flow-derived load. Studies related to atherosclerosis have demonstrated that chronic levels of low shear stress and variations in shear stress direction over the cardiac cycle, called multidirectional shear stress, both promote the expression of numerous atherogenic signaling molecules, including NF-κB, within endothelial cells leading to a dysfunctional phenotype.^11^,^22^,^34^ As a result, atherosclerotic plaques tend to localize to regions of the vasculature that experience these flow disturbances, such as within the inner curvature of arteries^29^ or near bifurcations.^18^ More recent studies have shown that introducing these flow disturbances within the arteries of hypercholesterolemic animals can induce the development of advanced atherosclerotic plaques.^7^,^27^

Despite many studies demonstrating the important relationship between shear stress and plaque development, it is still unclear what differences may exist in the vascular mechanical environment to promote the development of a stable advanced atherosclerotic plaque vs. one vulnerable to rupture. Towards this end, we recently hypothesized that the vessel wall mechanics may act in concert with the hemodynamics to promote different advanced plaque phenotypes.^26^ This hypothesis follows from the logic that a load (or deformation) derived from dilatation of the vessel wall due to blood pressure should be important for endothelial cell function (and, in the case of disturbances, dysfunction) similar to a load derived from the flow of blood. Indeed, several studies of normal endothelial cell function have shown that both shear stress and cyclical stretch can regulate the expression of mediators of vascular tone, proliferation, migration, angiogenesis, and the barrier function.^9^,^19^

In addition to regulating mediators of normal endothelial cell functions, normal cyclical stretch has also been shown to down-regulate pro-inflammatory mediators. For example, Korff *et al*.^16^ demonstrated that endothelial cells exposed to normal cyclical uniaxial stretch (reported as 15% membrane strain at 0.5 Hz) down-regulated expression of the leucocyte surface receptor CD40 compared to un-stretched controls and Liu *et al*.^20^ showed that cyclical equibiaxial stretch (6% strain at 1 Hz) attenuated the apoptotic effects of the cytokine TNF-α. Thus, physiological stretch of endothelial cells promotes an atheroprotective phenotype. On the other hand, related studies demonstrating that pathological or disturbed stretch can promote an atherogenic endothelial cell phenotype are scarce. In one recent study, Amaya *et al*.^1^ exposed cells to cyclical uniaxial stretch and pulsatile flow *in vitro* and showed that when stretch (8% strain at 1 Hz) and flow are 180° out-of-phase (compared to in phase), endothelial cells significantly up-regulated pro-atherogenic genes, including NF-κB. While this is an important finding, neither this study nor other previous studies have addressed how precise changes to normal stretch magnitude and direction of the endothelial cell might promote a pro-atherogenic phenotype, despite numerous studies demonstrating altered strain within regions of advanced atherosclerotic plaques.^4^,^14^,^36^

To address this need, we designed, built, and programmed a custom cell stretching device to mimic the complex vascular endothelial cell stretch environment *in vivo*. We examined whether low magnitudes of stretch and multidirectional stretch promote increased nuclear expression of the pro-atherogenic transcription factor NF-κB, compared to normal stretch. These disturbed stretch conditions are analogous to the most atherogenic disturbed flow profiles, namely low and multidirectional flow.

## Materials and Methods

### Cell Stretching Device

**Figure 1:**
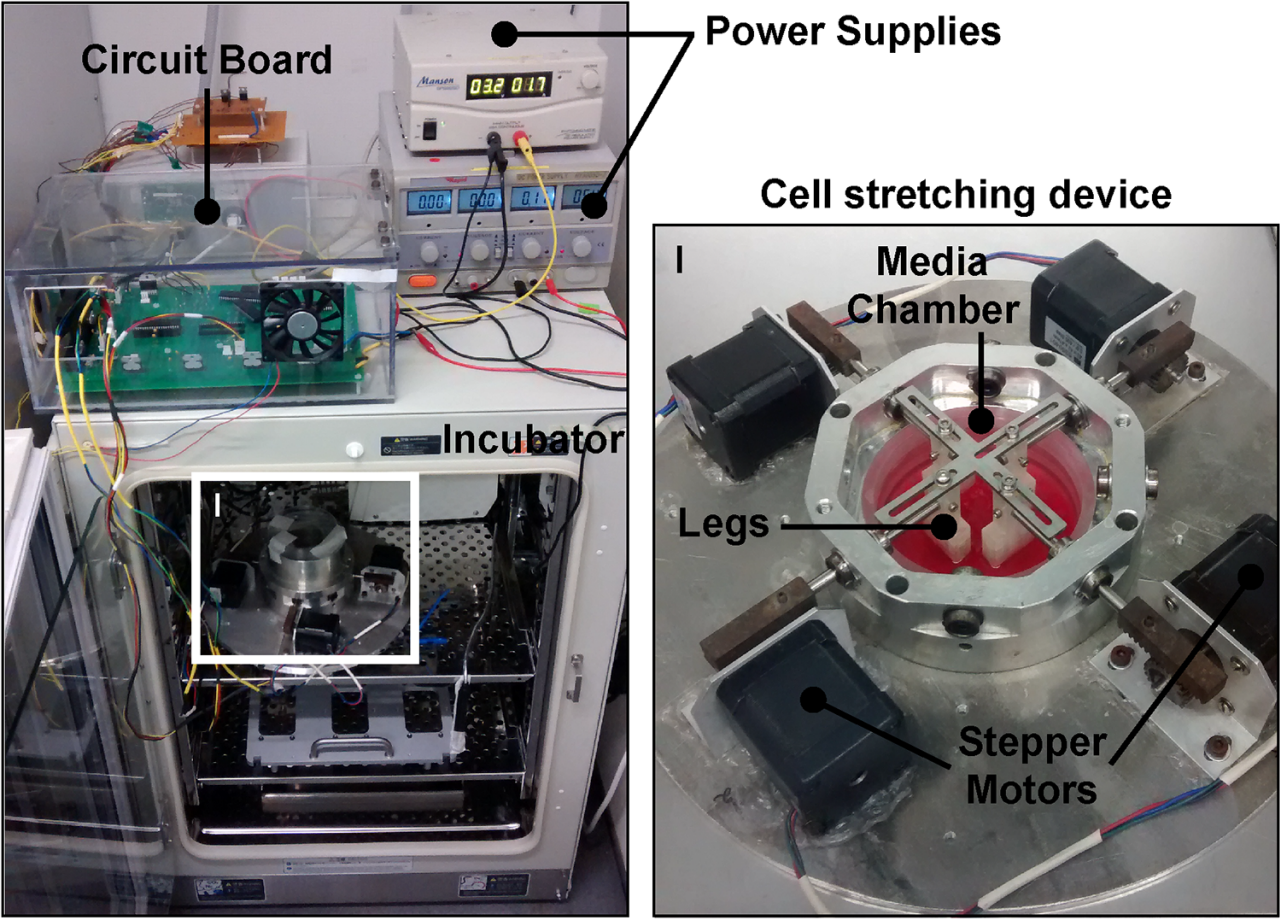
Image of the cell stretching device within the incubator attached by the four stepper motors to the electronic circuit board controller and power supplies. Inset (I) shows a close up of the stretching device where each of the four motors is attached by a stainless steel rod to a polycarbonate leg, which, in turn, is attached to the cell-seeded silicon membrane submerged in culture medium within the media chamber. Also shown is a steel cross-bar that is attached to the top of each leg, which allows the legs to be maintained in position from the time of seeding to placement in the stretching device for an experiment; the cross-bar is removed before the start of each experiment.

We designed, built, and programmed a custom cell stretching device that provided independent control over four high torque, 200-step stepper motors (RS Components, UK) in terms of displacement magnitude, direction, and frequency, which could be programmed to change over the course of the experiment. Each motor of the device was attached* via* a stainless steel rod to a polycarbonate leg, to which the cell substrate—a 0.25 mm thick silicon membrane (Silex Silicones, UK)—was attached by a steel plate screwed into the bottom of each leg (Fig. 1). The stepper motors were controlled by a custom-made printed circuit board using a PIC16F887 family microcontroller. The microcontroller was programmed in the PIC C language (Custom Computer Services, USA). The program was compiled in MPLAB and imported into the microcontroller using the PICKit3 programmer (Microchip, USA). A different microcontroller code was developed for each experimental protocol.

We used our custom cell stretching device to replicate the stretch environment of the arterial endothelial cell *in vivo*. The normal stretch condition first imposed a pre-stretch (or residual stretch) of 1.06 (equivalent to 6.18% Green strain) in the first direction of the membrane (representing the circumferential direction of the vessel) and 1.10 (10.50% Green strain) in the second (orthogonal) direction (representing the axial direction of the vessel; based on Masson *et al*.^21^), and then began cyclical stretching in the first direction between 1.06 and 1.14 (14.98% Green strain; based on Morrison *et al*.^23^) while holding the second direction static. Three disturbed stretch conditions were then examined. The first condition imposed either a low magnitude of cyclical stretch that was chosen to be 1.02 (2.02% Green strain) or no stretch, which were motivated by the analogous atherogenic flow condition low shear stress (the latter taken to the stagnation point). The endothelial cell response to low cyclical stretch was compared to normal cyclical stretch with and without the inclusion of pre-stretch of the membrane. The second disturbed stretch condition assessed the effects of multidirectional stretch by altering the cyclical stretch direction every 1 s and using either the normal (1.08) or low (1.02) cyclical stretch magnitude (pre-stretch was excluded to maximize the pro-atherogenic response). This stretch condition was motivated by atherogenic multidirectional flow. The third disturbed stretch condition attempted to maximize the pro-atherogenic endothelial cell response to stretch by randomly altering both the stretch magnitude (using the magnitude from either the normal or low cyclical stretch conditions) and direction (using either the first or second (orthogonal) direction) every 30 min. This period was chosen as NF-κB expression within cells peaks at 30 min.^15^ Finally, TNF-α (10 ng/ml) was added to the culture media of un-stretched cells for 30 min as a positive control. All experimental stretch conditions (see Table 1 for summary) were performed over 6 h and cyclical stretching was done at a frequency of 1 Hz.

**Table 1:** Summary of experimental stretch conditions.

	Condition	Pre-stretch	Cyclical stretch
1	Normal stretch	CS: 1.06	CS: 1.06 to 1.14
		AS: 1.10	
2	Normal stretch,	None	CS: 1.00 to 1.08
	no pre-stretch		
3	Low stretch	CS: 1.06	CS: 1.06 to 1.08
		AS: 1.10	
4	Low stretch,	None	CS: 1.00 to 1.02
	no pre-stretch		
5	No stretch	None	None
6	Multidirectional	None	CS or AS (altered every 1 sec):
	normal stretch		1.08
7	Multidirectional	None	CS or AS (altered every 1 sec):
	low stretch		1.02
8	Varied stretch	None	CS or AS (altered every 30 min):
			1.02 or 1.08 (altered every 30 min)
9	TNF-α (10 ng/ml) (positive control)	None	None

*CS* circumferential stretch, *AS* axial stretch

### Calibration

The relationship between motor rod displacement and deformation of the membrane within the region of cell attachment was determined by a calibration. Three membranes were placed within the device and five ink points were placed in an X-pattern within each of the four quadrants of the membrane where cells would normally be seeded. Each membrane was uniaxially stretched through a single loading and unloading cycle up to 2.7 mm of rod displacement (5.4 mm total displacement of the opposing rods) and images were acquired at 11 steps per cycle. Images were thresholded and the centroid of each ink mark was determined in ImageJ.^30^ The deformation gradient tensor, *F*, was quantified from marker displacements within each set of five points, which was then used to compute Green strain, *E*,* via* $E = \frac{1}{2}\left({F^{T} \cdot F - I} \right)$ where *I* is the identity tensor.^25^ Green strain is properly insensitive to rigid body motion and related to stretch, *λ*, in one-dimension* via* $E = \frac{1}{2}\left({{\lambda}^{2} - 1} \right)$. Each component of the Green strain tensor was averaged over the four quadrants of the membrane where cells are seeded and a linear regression was used to determine the relationship with membrane displacement.

### Fluid–Structure Interaction Modelling

To evaluate the magnitude and uniformity of strain and shear stress (due to media flow) distributions over the stretched membrane, a 3-D fluid–structure interaction (FSI) model of the cell stretching device was developed in Abaqus (v6.14, Dassault Systèmes, France). The model was composed of steel motor rods, polycarbonate legs, steel fixing plates, the silicon cell substrate, and an acrylic media chamber. All materials were modeled as linear elastic with material properties based on the manufacturers specifications (Table 2). The cell culture media was defined as an incompressible Newtonian fluid similar to saline solution with a density of 1050 kg/m^3^ and viscosity of 0.0035 Pa·s.

**Table 2:** Material parameters for model components.

Material	Elastic modulus (GPa)	Poisson’s ratio (ND)	Density (kg/m^**3**^ **)**
Acrylic	3.20	0.39	1190
Polycarbonate	2.50	0.39	1190
Silicon rubber	1.69 × 10^−3^	0.39	2330
Steel	200	0.30	7900

The critical components of the model were the cell substrate and culture media, which were meshed using 13,848 and 185,085 8-node linear hexahedral elements, respectively. A convergence test was performed by iteratively running simulations within increasing mesh density of the membrane and media. The model was considered converged when membrane strain and shear stress differed by less than 0.1 and 0.2%, respectively.

All simulations were performed using Abaqus/CFD and Abaqus/Standard modules in a FSI co-simulation. Each simulation replicated the normal (uniaxial) cyclical stretch condition without pre-stretch by prescribing a maximum displacement of two opposing rods of 1.57 mm per rod (in opposite directions) at 1 Hz (rods in the orthogonal direction remained stationary). The interface layer of the model was defined as the contact between the culture media and the cell substrate and media chamber. Every simulation was run over four cycles to evaluate finite strain and shear stress acting on the cell substrate (these metrics were within 0.1 and 0.6%, respectively, between the third and fourth cycles, demonstrating periodic convergence).

### Cell Culture

The EA.hy926 cell line, which is the most widely used and thoroughly characterized immortalized endothelial cell line,^5^ was used for all experiments herein. EA.hy926 cells were maintained in Dulbecco’s Modified Eagle’s medium (DMEM) supplemented with 10% (v/v) fetal bovine serum (FBS), 2.5% l-glutamine (2.5 mmol/L), 1% penicillin (100 IU/ml), and 1% streptomycin (100 IU/ml) within a humidified incubator at 37 °C and 5% CO_2_. Cells were sub-cultured prior to confluence by brief exposure to trypsin/EDTA (0.1% /0.02% in PBS) and seeded at a ratio of 1:6 (all chemicals were obtained from Sigma-Aldrich, UK, unless otherwise indicated).

Prior to cell seeding, each silicon membrane was covalently coated with 0.1% gelatin from bovine skin to facilitate cell attachment and lightly pre-stretched (~ 1%) in a polycarbonate ring device.^6^ Four polycarbonate legs of the stretching device were then attached to the pre-stretched membrane with M5 screws into the threaded holes in the bottom of the legs. The membrane was then cut free of the ring device and four cloning rings (each with a cross-sectional area of 0.5 cm^2^) were attached to the bottom (gelatine coated) surface of the membrane with silicon grease. Cells were suspended in 200 µl of media and seeded into each cloning ring at a density of 90,000 cells/cm^2^; thus, each membrane yielded up to four cell monolayers. After 4 h within an incubator, the cloning rings were removed and the cell-seeded membrane was entirely submerged in media and cultured for another 2 d before performing the stretching experiments.

### NF-κB Staining and Imaging

At the end of each stretching experiment, media was replaced with 4% paraformaldehyde in PBS while continuing stretch for another 5 min. The membrane was then removed from the device and cells were immunostained for NF-κB expression as follows. Cells were permeabilized with 0.1% triton × 100 in PBS for 5 min and then incubated with blocking buffer (0.5% w/v bovine serum albumin in PBS) for 1 h to prevent non-specific binding. NF-κB was targeted by incubating cells overnight at 4 °C with a rabbit polyclonal primary antibody that targeted the p65 domain (Santa-Cruz, UK), which was diluted 1:100 in blocking buffer. Detection was done using Alexa Fluor 594 goat anti-rabbit fluorescent secondary antibody (Santa-Cruz) diluted 1:250 in blocking buffer with an incubation period of 1 h at room temperature. DAPI was used as a nuclear counter-stain at a concentration of 2.5 μg/mL in PBS and an incubation period of 15 min. Finally, cell monolayers were mounted onto slides using Ibidi mounting medium for fluorescent microscopy (VWR, UK).

Mounted cell monolayers were imaged with a Zeiss PALM laser capture microscope at ×20 magnification. NF-κB was imaged with an excitation of 560/40 nm and emission was collected with a Texas red filter at 630/75 nm. DAPI was imaged with an excitation of 405/30 nm and collected with a DAPI filter at 450/40 nm. For each cell monolayer, images from two to four microscopic fields were acquired along a circumferential arc at a radial position of ~3 mm from the center of the monolayer.

### Image Analysis and Quantifying NF-κB Activation

The primary readout in this study is the ratio of NF-κB in the cell nucleus to the cytoplasm under different stretch conditions, wherein higher values of this ratio equate to higher levels of nuclear NF-κB. This approach accounts for possible differences in fluorescence intensity between samples, although cytoplasmic NF-κB levels may also increase with nuclear translocation meaning that results represent a conservative estimate of nuclear NF-κB.

To obtain this readout, cells were segmented from each image using a custom Matlab (R2012, Mathworks, USA) program as follows. First, a representative subset of cell nuclei evenly distributed over the field of view were randomly selected from the DAPI image. The boundary of each cell was then segmented from the corresponding NF-κB image with the nuclear portion of the stain removed (based on the DAPI segmentation) to mask the user from qualitatively evaluating the amount of nuclear NF-κB staining within the selected cells. Overall, 21.4 ± 5.3% of all cells were randomly selected for segmentation within each image, which equated to a mean of 19 cells per image and 57 cells over the three images typically acquired per monolayer. The ratio of NF-κB stain intensity in the cell nucleus to the cytoplasm was then computed for each segmented cell and averaged over all cells segmented within all images of a monolayer. Thus, herein, *n* designates the number of cell monolayers analyzed per experimental condition. The mean of this ratio over all monolayers of a given experimental condition is reported, scaled by the mean within cell monolayers exposed to the normal stretch condition. To validate that the subset of selected cells was representative of the overall population of the monolayer, a convergence test was performed to examine the mean NF-κB intensity ratio over increasing numbers of cells (e.g., the mean was assessed over two cells, three cells, etc.) and intensity ratio values were found to converge after averaging over 20 to 30 cells per monolayer (which is below the mean of 57 cells segmented per monolayer). The mean error associated with convergence over all monolayers (*n* = 43 monolayers over all experimental conditions) was 0.57 ± 0.36%.

### Statistical Analysis

Data are reported as mean ± SD. A Shapiro–Wilk test was used to determine that the distributions of all data were not statistically different from a normal distribution. A one-way ANOVA was then used to evaluate statistical significance and a posthoc multiple comparisons test was employed with a Tukey–Kramer correction to explore the significance of individual comparisons. *P* < 0.05 was used as the threshold for statistical significance. All statistical analyses were performed in Matlab.

## Results

**Figure 2:**
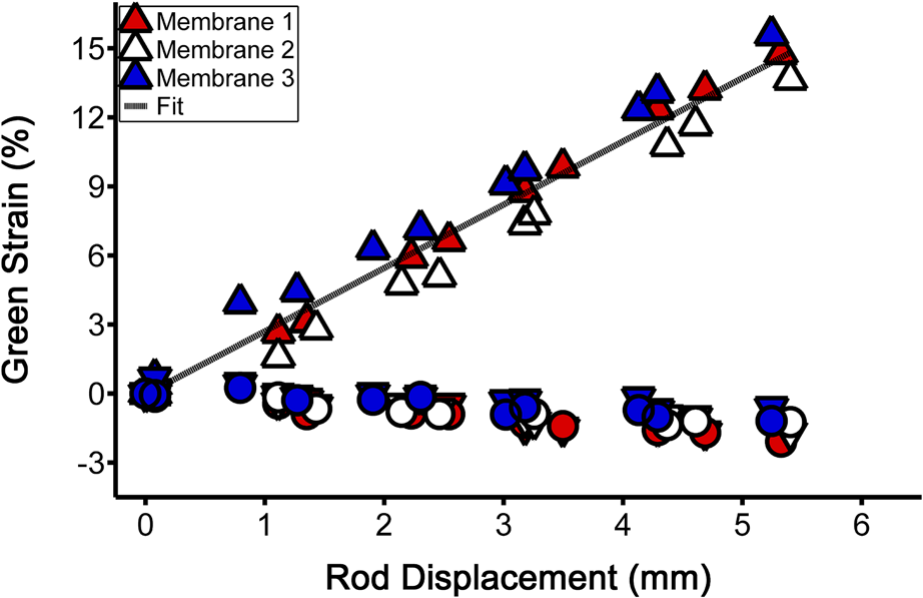
Calibration of the silicon membranes (*n* = 3) was performed to determine the relationship between rod displacement (mm) and Green strain (%). Uniaxial extension tests demonstrated a linear elastic behavior of the membrane with a mean slope of 2.77 ± 0.17% Green strain per mm of total (absolute) displacement of the opposing rods, as determined by a linear regression (indicated by the finely dotted black line). Triangles facing upwards indicate the primary stretching direction, those facing downwards indicate the orthogonal stationary direction, and circles indicate the direction of shear strain (for conciseness, the latter two symbols are not included in the legend).

**Figure 3:**
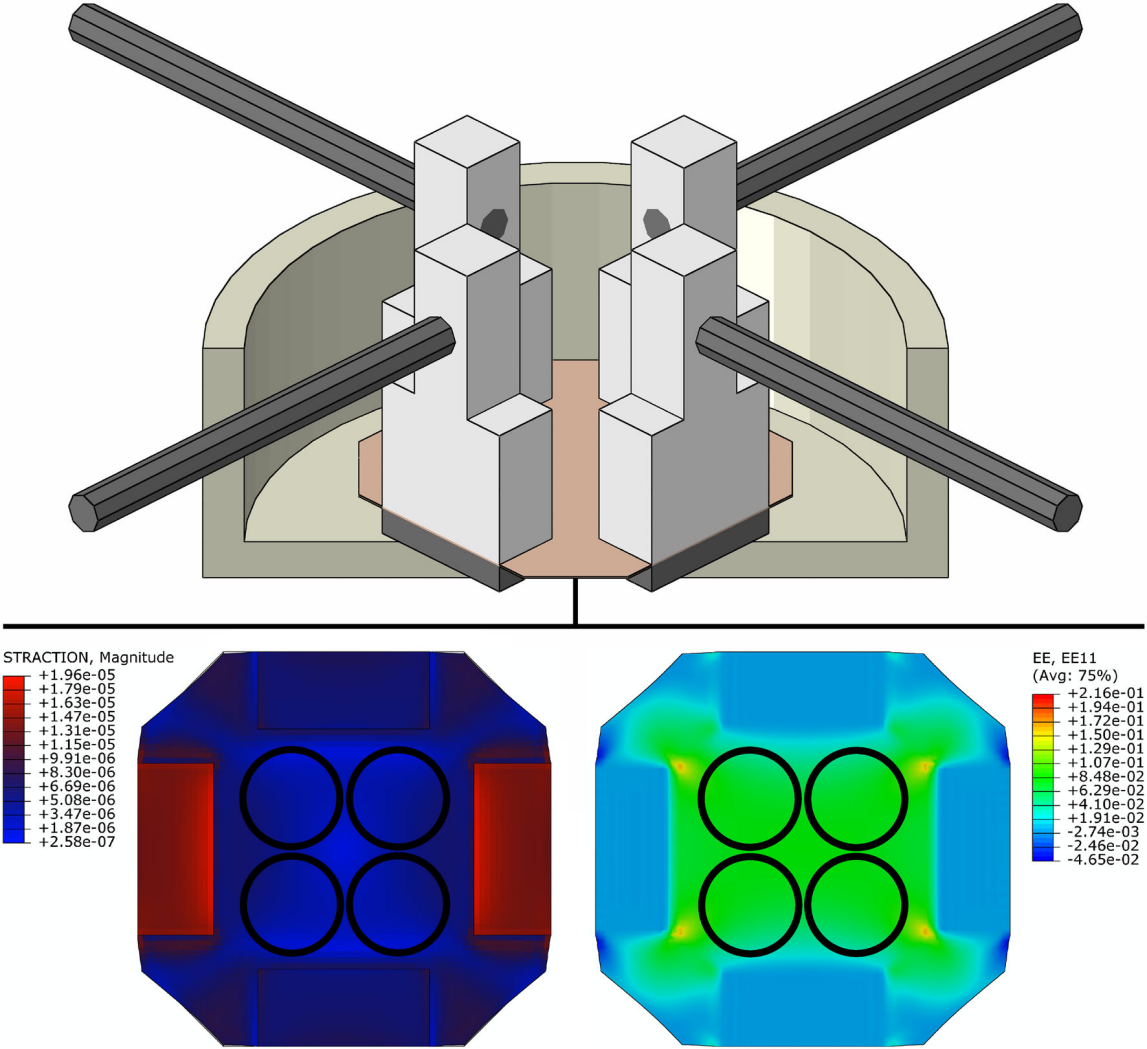
(Top) FSI model of the stretching device, composed of the motor rods (dark grey) attached to the polycarbonate legs (light grey) which, in turn, are attached to the silicon membrane (flesh color) and secured with a steel plate (dark grey). This assembly sits in a media chamber (shown as a cutaway with the fluid domain removed). (Bottom) Distributions of shear stress (left, labeled as STRACTION; kPa) and finite strain (right, labeled as EE) over the cell-seeded side of the membrane at the maximum rod displacement used in the experimental conditions. The magnitude of shear stress was far below physiologic and strain was nearly uniform, particularly in the region where the four cloning rings are placed to seed the cells (indicated by black circles).

We began by validating the magnitude and distribution of strain and shear stress imposed onto the cell-seeded membrane by the stretching device. Calibration of the membrane by uniaxial extension tests showed a linear relationship between rod displacement and membrane strain within the region of cell attachment, wherein the mean slope of the regression line was 2.77 ± 0.17% Green strain (*n* = 3 membranes; equivalent to a stretch of 1.027) per mm of rod displacement (Fig. 2). Uniaxial extension tests performed on membranes in the orthogonal direction showed no statistical differences in behavior (*p* = 0.41). FSI modeling further demonstrated that membrane displacement during uniaxial extension caused a nearly uniform distribution of strain within the region of cell attachment (Fig. 3). In addition, the FSI model showed that the shear stress imposed onto the cells as the membrane is cyclically stretched at 1 Hz while immersed in media is a maximum of 0.006 Pa, which is far below physiological values of approximately 1.5 Pa and thus negligible in this system.

**Figure 4:**
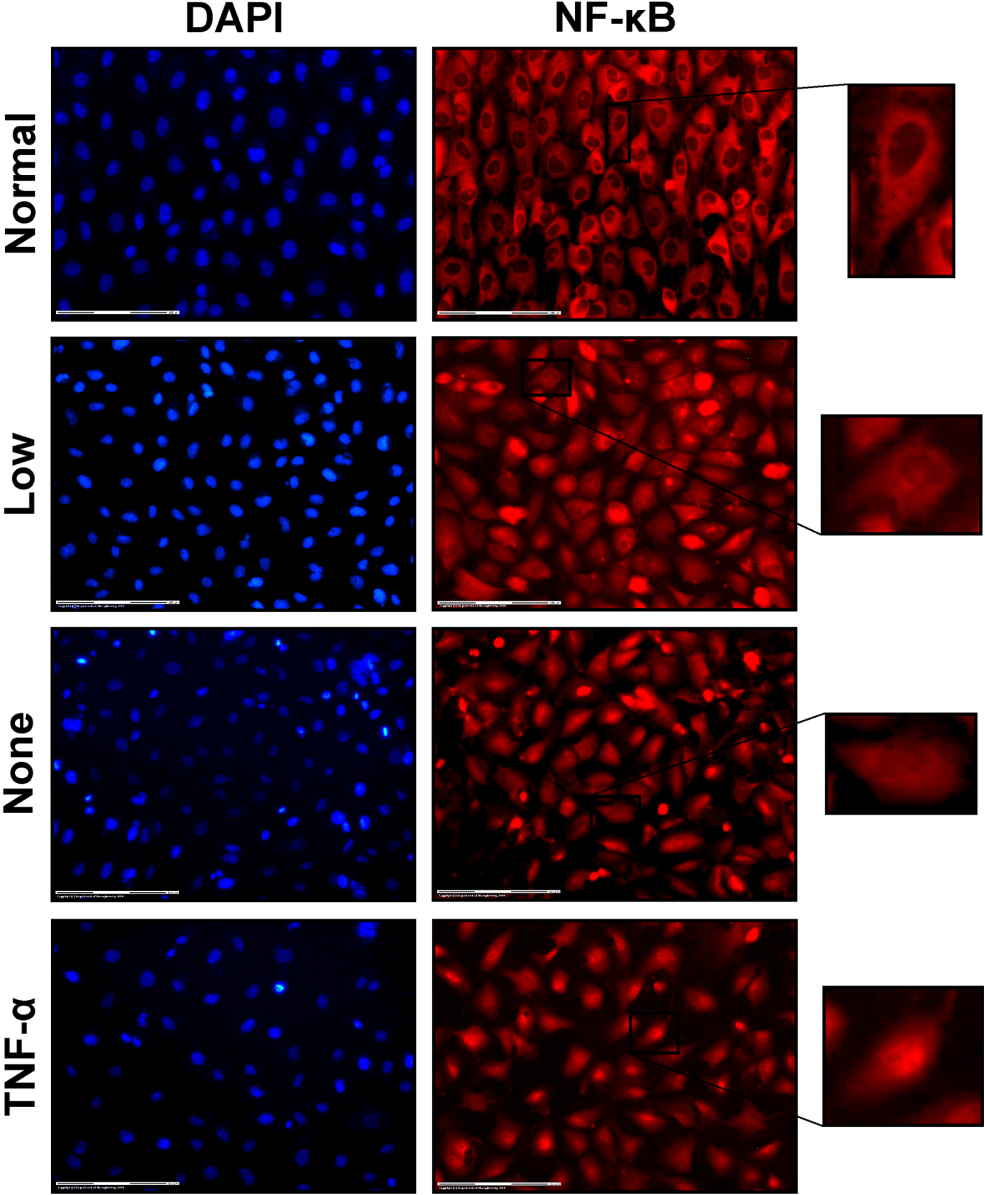
Representative images of EA.hy926 endothelial cells with DAPI nuclear (left column) and NF-κB (right column) stains. Compared to cells exposed to normal stretch (first row), cells exposed to a low magnitude of stretch (second row) or no stretch (third row) exhibited higher nuclear expression of NF-κB. TNF-α is shown as a positive control (fourth row). A representative cell from each NF-κB image is magnified to better illustrate nuclear expression. Scale bars are 150 *µ*m.

**Figure 5:**
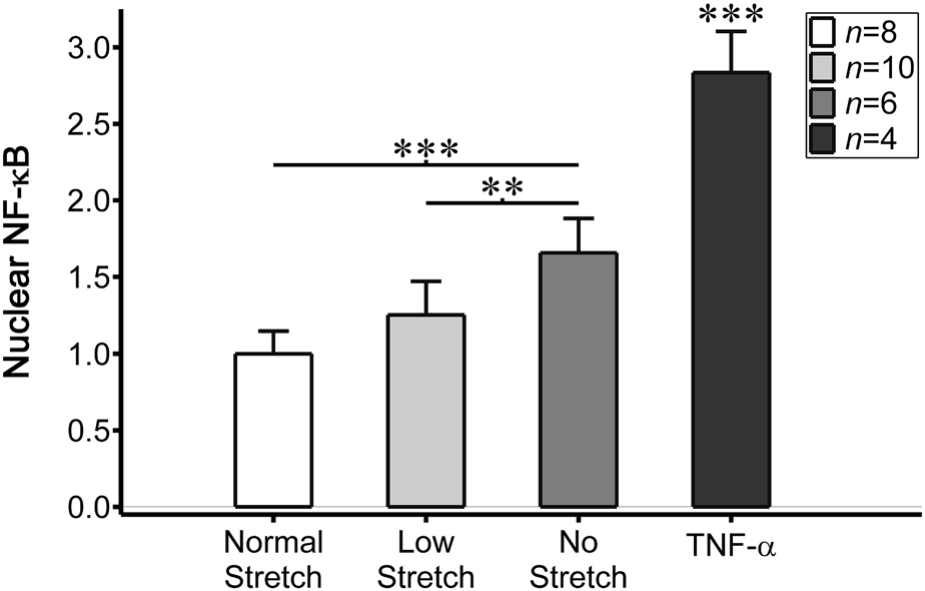
Nuclear NF-κB (quantified as the ratio of nuclear to cytoplasmic NF-κB and scaled by values within cells exposed to normal stretch) within endothelial cells exposed to normal cyclical stretch (white bar, *n* = 8 cell monolayers), low cyclical stretch (light grey bar, *n* = 10), no stretch (dark grey bar, *n* = 6), or TNF-α (near-black bar, *n* = 4). Cells exposed to lower cyclical stretch demonstrated higher levels of nuclear NF-κB, although TNF-α promoted the highest expression (statistically higher than all stretch conditions). Statistically significant differences are indicated by **p < 0.01 and ***p < 0.001. Error bars indicate ± SD.

The main aim of our study was to test the hypothesis that disturbed stretch conditions, analogous to known atherogenic flow profiles, would promote a pro-atherogenic endothelial cell phenotype corresponding to higher levels of nuclear NF-κB (quantified as the ratio of nuclear to cytoplasmic NF-κB). We first compared the nuclear NF-κB levels in endothelial cell monolayers exposed to normal (physiological) cyclical stretch to those exposed to a low magnitude of cyclical stretch or no stretch (Fig. 4). All data were scaled to the nuclear NF-κB levels exhibited by endothelial cells exposed to normal stretch, which, after scaling, was 1.00 ± 0.15 (*n* = 8 cell monolayers). Nuclear NF-κB expression increased in endothelial cells as stretch magnitude decreased with mean values of 1.25 ± 0.22 in cells exposed to low stretch (*n* = 10; *p* = 0.09 compared to normal stretch) and 1.66 ± 0.22 in cells exposed to no stretch (*n* = 6; *p* < 0.001 compared to normal and *p* < 0.01 compared to low stretch conditions). As a positive control, TNF-alpha was added to un-stretched monolayers (*n* = 4), which demonstrated significantly higher values of nuclear NF-κB of 2.83 ± 0.27 compared to endothelial cells exposed to the normal, low, or no stretch conditions (*p* < 0.001; Fig. 5).

**Figure 6:**
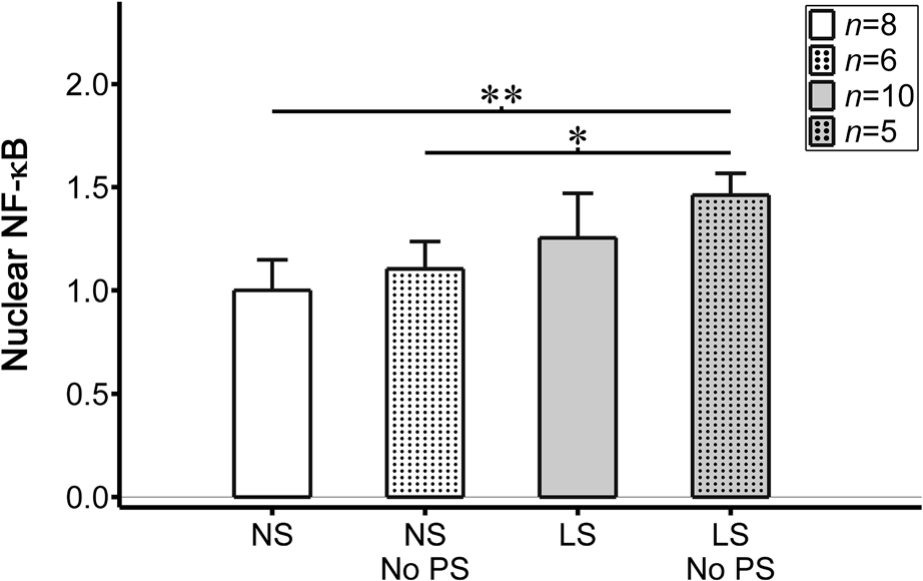
Nuclear NF-κB (quantified as the ratio of nuclear to cytoplasmic NF-κB and scaled by values within cells exposed to normal stretch) within endothelial cells exposed to normal or low cyclical uniaxial stretch (NS and LS, respectively) with (*n* = 8 and 10 monolayers for NS and LS, respectively; indicated by solid bars) or without (*n* = 6 and 5 monolayers, respectively; indicated by dotted bars) the non-uniform biaxial pre-stretch (PS). Removal of pre-stretch led to an increase in nuclear NF-κB within the cell monolayers, though it was not statistically significant. Other comparisons are indicated as statistically different by *p < 0.05 and **p < 0.01. Error bars indicate ± SD.

To evaluate the importance of static pre-stretch of the membrane prior to cyclical cell stretching in determining the difference in nuclear NF-κB expression by endothelial cells exposed to normal vs. low stretch conditions, we examined both of these stretch conditions without pre-stretch. In the normal stretch condition, values of nuclear NF-κB were 1.10 ± 0.13 without pre-stretch (*n* = 6) vs. 1.00 ± 0.15 with pre-stretch (*n* = 8; *p* = 0.96). In the low stretch condition, values were 1.46 ± 0.11 (*n* = 5) vs. 1.25 ± 0.22 (*n* = 10; *p* = 0.47), respectively (Fig. 6). Thus, removing pre-stretch of the membranes caused an increase in nuclear NF-κB expression within the endothelial cells, but it was not statistically higher than cells exposed to the respective condition with pre-stretch.

**Figure 7:**
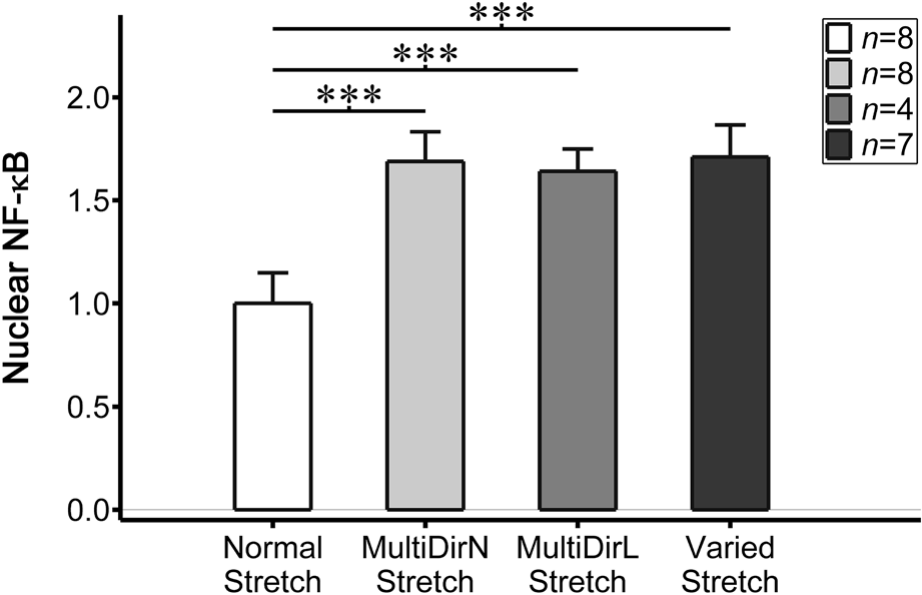
Nuclear NF-κB (quantified as the ratio of nuclear to cytoplasmic NF-κB and scaled by values within cells exposed to normal stretch) within endothelial cells exposed to normal stretch (white bar, *n* = 8 cell monolayers), multidirectional stretch with a normal magnitude (light grey bar, *n* = 8), multidirectional stretch with a low magnitude (dark grey bar, *n* = 4), and varied stretch (near-black bar, *n* = 7). Statistically significant differences are indicated by ***p < 0.001. Error bars indicate ± SD.

Next, we examined nuclear NF-κB expression within endothelial cells exposed to a second disturbed stretch condition, multidirectional stretch, which was performed using cyclical stretch magnitudes from either the normal or low stretch conditions. Endothelial cells exposed to multidirectional cyclical stretch with a normal stretch magnitude exhibited mean nuclear NF-κB expression levels of 1.69 ± 0.14 (*n* = 8), which was statistically higher than levels in cells exposed to the normal stretch condition (*p* < 0.001). A similar increase in nuclear NF-κB was also observed in endothelial cells exposed to multidirectional cyclical stretch with a low stretch magnitude wherein mean expression levels were 1.64 ± 0.11 (*n* = 4), which was also statistically higher than levels in cells exposed to normal stretch (*p* < 0.001; Fig. 7). Nuclear NF-κB expression levels in cells exposed to either of the multidirectional stretch conditions were also statistically higher than those exhibited by cells exposed to the low stretch condition (*p* < 0.001).

Finally, we explored a theoretical condition that randomly varied the cyclical stretch magnitude and direction every 30 min to examine a possible maximal pro-atherogenic endothelial cell response to disturbed stretch. Endothelial cells exposed to this varied stretch condition exhibited nuclear NF-κB levels of 1.71 ± 0.16 (*n* = 7), which was only slightly higher than (and not statistically different from) expression levels in cells exposed to either multidirectional stretch condition (Fig. 7). Accordingly, these expression levels were also statistically higher than the levels for cells exposed to both normal (*p* < 0.001) and low (*p* < 0.001) stretch. Neither the varied nor multidirectional stretch conditions were statistically different from the no stretch condition.

## Discussion

Although certain blood flow profiles within the artery are known to promote a pro-atherogenic endothelial cell phenotype, relatively few studies have examined the influence of the vessel wall mechanics. Our results herein are the first to demonstrate that decreasing levels of stretch magnitude from normal and multidirectional stretch both promote increased nuclear expression of the pro-atherogenic transcription factor NF-κB. Each of these two disturbed stretch conditions was motivated by an analogous atherogenic flow profile, low flow and multidirectional (or oscillatory) flow, which suggests that disturbed vessel wall stretch can be atherogenic similar to disturbed flow. We also considered static pre-stretch of the membrane in promoting endothelial cell quiescence, which had not been examined by previous investigators. Cyclical stretching without pre-stretch demonstrated an increase in nuclear NF-κB expression compared to cells exposed to the same cyclical stretch condition with pre-stretch. However, this increase was not statistically significant, which demonstrates that the cyclical aspect of the wall stretch environment is the most important.

Our work builds on previous studies showing that stretch, in general, can up-regulate many pro-inflammatory signaling molecules, including: NF-κB,^10^ monocyte chemotactic protein-1 (MCP-1),^35^ intercellular adhesion molecule-1 (ICAM-1),^8^ matrix metalloproteinases (MMPs),^33^ and c-jun N-terminal kinase (JNK, a member of the mitogen activated protein kinase family).^3^ However, whereas our study used a custom cell stretching device to impose a precisely controlled and uniform strain over the membrane, these studies are limited by the use of a commercial device (Flexcell FX-2000) that imposed a highly non-uniform strain over the membrane (typically 0% at the center to 24% at the periphery). Nevertheless, these studies showed an up-regulation of pro-inflammatory signaling molecules with higher levels of stretch. Later studies continued to examine the effects of high stretch, but with an updated Flexcell system (FX-3000 and FX-4000) that employed loading posts for more uniform and equibiaxial stretch (although still with inhomogeneities^13^). These studies similarly demonstrated that high cyclical equibiaxial stretch (reported as 20% strain at 1 Hz) potentiated apoptosis within endothelial cells due to increased production of reactive oxygen species.^17^,^20^ In addition to studies of vascular endothelial cells, studies of pulmonary endothelial cells have also shown that higher than normal stretch levels (e.g., ventilator-induced) promote a pro-inflammatory phenotype.^12^,^28^

Only one other study has examined low stretch. Thacher *et al*.^31^ demonstrated that isolated endothelial cells exposed to low cyclical circumferential stretch (1% strain at 1 Hz) decreased expression of eNOS, a molecule of endothelial cell functionality, compared to normal stretch (5% strain at 1 Hz) and, within isolated porcine carotid arteries examined* ex vivo*, low cyclical stretch caused increased reactive oxygen species production. Collectively, our results and those previously reported demonstrate that deviations from normal in cyclical stretch magnitude, low or high, can promote a pro-inflammatory endothelial cell phenotype.

One important consideration is the actual stretch environment of the endothelial cell *in vivo*. In a healthy vessel, the endothelium experiences a maximum cyclical circumferential stretch of between 1.05 and 1.11 (mean of 1.08)^21^,^23^ plus a residual stretch of approximately 1.06 in the circumferential direction and 1.10 in the axial direction.^21^ With age, arteries stiffen due to a lesser composition of elastin^32^ and glycosylation of major structural proteins like collagen,^2^ which is strongly associated with the development of atherosclerosis.^2^,^23^,^24^ Higher stiffness of the artery would result in a lower stretch, which, in addition to the analogous disturbed flow condition of low flow, motivated our examination of low stretch in promoting a pro-atherogenic endothelial cell phenotype. However, it remains unclear whether endothelial cells experience low or high stretch over the development of an atherosclerotic plaque, particularly in the more advanced stages.

Several studies have attempted to quantify the distribution of strain within advanced plaques from either histological sections or *in vivo* images and most have shown that, particularly within advanced plaques, the region of the fibrous cap experiences high strain.^14^,^36^ These findings combined with those above suggest that endothelial cells within diseased vessels may initially experience low strain due to vessel stiffening which transitions to high strain as a (thin) fibrous cap forms towards the end of advanced plaque development. However, these studies neither consider residual strain of the vessel nor relate strain values to those found in healthy vessels often leading to “high strain” magnitudes far below normal.^4^,^14^,^36^ In addition, several studies have reported measures of strain in the radial direction instead of the circumferential direction, which is the primary load bearing direction of the vessel.^4^,^14^ Overall, these limitations make it difficult to ascertain whether endothelial cells actually experience high strain in the final stages of advanced plaque development. An alternative possibility is that the fibrotic cap of an advanced plaque contains regions of both low and high strain relative to normal, which has been reported previously.^4^

In addition to changes in stretch magnitude, it is possible that certain arteries may also experience variations in the direction of wall stretch over the cardiac cycle as occurs with multidirectional flow. Although to our knowledge no study has examined changes in stretch direction within arteries, we hypothesize that multidirectional stretch could occur particularly within coronary arteries as these vessels are exposed to distension due to blood pressure and bending/torsion due to the contracting heart, which likely deform the vessel in different directions. Multidirectional stretch could also conceivably occur in the advanced stages of atherosclerosis, particularly in complex parts of the vasculature such as near bifurcations. Additional modeling studies, particularly using a fluid–structure interaction approach, are needed to explore changes in vessel wall stretch over the cardiac cycle. In the meantime, our results are the first to demonstrate a role for multidirectional stretch in promoting a pro-atherogenic endothelial cell phenotype.

### Conclusion

We developed a custom cell stretching device that can impose precise levels of static and cyclical stretch to examine whether low and multidirectional stretch of endothelial cells promotes nuclear expression of NF-κB, compared to normal stretch. These stretch conditions were motivated by analogous atherogenic flow profiles, low and multidirectional flow. We found that both stretch conditions up-regulated nuclear NF-κB, which suggests that disturbances to wall stretch can be atherogenic similar to disturbed flow. Complimenting flow studies, this finding provides a more complete picture of the potential role of disturbed vessel biomechanics in promoting a dysfunctional endothelial cell phenotype during the development of advanced atherosclerotic plaques.

## References

[1] Amaya R, Pierides A, Tarbell JM (2015). The interaction between fluid wall shear stress and solid circumferential strain affects endothelial gene expression. PLoS ONE.

[2] Aronson D (2003). Cross-linking of glycated collagen in the pathogenesis of arterial and myocardial stiffening of aging and diabetes. J. Hypertens..

[3] Azuma N, Duzgun SA, Ikeda M, Kito H, Akasaka N, Sasajima T, Sumpio BE (2000). Endothelial cell response to different mechanical forces. J. Vasc. Surg..

[4] Baldewsing RA, Schaar JA, Mastik F, Oomens CW, van der Steen AF (2005). Assessment of vulnerable plaque composition by matching the deformation of a parametric plaque model to measured plaque deformation. IEEE Trans. Med. Imaging.

[5] Bouis D, Hospers GA, Meijer C, Molema G, Mulder NH (2001). Endothelium in vitro: a review of human vascular endothelial cell lines for blood vessel-related research. Angiogenesis.

[6] Braakman ST, Pedrigi RM, Read AT, Smith JA, Stamer WD, Ethier CR, Overby DR (2014). Biomechanical strain as a trigger for pore formation in Schlemm’s canal endothelial cells. Exp. Eye Res..

[7] Cheng C, Tempel D, van Haperen R, van der Baan A, Grosveld F, Daemen MJ, Krams R, de Crom R (2006). Atherosclerotic lesion size and vulnerability are determined by patterns of fluid shear stress. Circulation.

[8] Cheng JJ, Wung BS, Chao YJ, Wang DL (1996). Cyclic strain enhances adhesion of monocytes to endothelial cells by increasing intercellular adhesion molecule-1 expression. Hypertension.

[9] Cummins PM, von Offenberg Sweeney N, Killeen MT, Birney YA, Redmond EM, Cahill PA (2007). Cyclic strain-mediated matrix metalloproteinase regulation within the vascular endothelium: a force to be reckoned with. Am. J. Physiol. Heart Circ. Physiol..

[10] Du W, Mills I, Sumpio BE (1995). Cyclic strain causes heterogeneous induction of transcription factors, AP-1, CRE binding protein and NF-kB, in endothelial cells: species and vascular bed diversity. J. Biomech..

[11] Frueh J, Maimari N, Homma T, Bovens SM, Pedrigi RM, Towhidi L, Krams R (2013). Systems biology of the functional and dysfunctional endothelium. Cardiovasc. Res..

[12] Gawlak G, Son S, Tian Y, O’Donnell JJ, Birukov KG, Birukova AA (2016). Chronic high magnitude cyclic stretch stimulates EC inflammatory response via VEGF Receptor 2 dependent mechanism. Am. J. Physiol. Lung Cell Mol. Physiol..

[13] Geest JPV, Di Martino ES, Vorp DA (2004). An analysis of the complete strain field within Flexercell(TM) membranes. J. Biomech..

[14] Gijsen FJ, Wentzel JJ, Thury A, Mastik F, Schaar JA, Schuurbiers JC, Slager CJ, van der Giessen WJ, de Feyter PJ, van der Steen AF, Serruys PW (2008). Strain distribution over plaques in human coronary arteries relates to shear stress. Am. J. Physiol. Heart Circ. Physiol..

[15] Hoffmann A, Levchenko A, Scott ML, Baltimore D (2002). The IkappaB-NF-kappaB signaling module: temporal control and selective gene activation. Science.

[16] Korff T, Aufgebauer K, Hecker M (2007). Cyclic stretch controls the expression of CD40 in endothelial cells by changing their transforming growth factor-beta1 response. Circulation.

[17] Kou B, Zhang J, Singer DR (2009). Effects of cyclic strain on endothelial cell apoptosis and tubulogenesis are dependent on ROS production via NAD(P)H subunit p22phox. Microvasc. Res..

[18] Ku DN, Giddens DP, Zarins CK, Glagov S (1985). Pulsatile flow and atherosclerosis in the human carotid bifurcation. Positive correlation between plaque location and low oscillating shear stress. Arteriosclerosis.

[19] Li YS, Haga JH, Chien S (2005). Molecular basis of the effects of shear stress on vascular endothelial cells. J. Biomech..

[20] Liu XM, Ensenat D, Wang H, Schafer AI, Durante W (2003). Physiologic cyclic stretch inhibits apoptosis in vascular endothelium. FEBS Lett..

[21] Masson I, Boutouyrie P, Laurent S, Humphrey JD, Zidi M (2008). Characterization of arterial wall mechanical behavior and stresses from human clinical data. J. Biomech..

[22] Mohan S, Koyoma K, Thangasamy A, Nakano H, Glickman RD, Mohan N (2007). Low shear stress preferentially enhances IKK activity through selective sources of ROS for persistent activation of NF-kappaB in endothelial cells. Am. J. Physiol. Cell Physiol..

[23] Morrison TM, Choi G, Zarins CK, Taylor CA (2009). Circumferential and longitudinal cyclic strain of the human thoracic aorta: age-related changes. J. Vasc. Surg..

[24] Palombo C, Kozakova M (2016). Arterial stiffness, atherosclerosis and cardiovascular risk: pathophysiologic mechanisms and emerging clinical indications. Vascul. Pharmacol..

[25] Pedrigi RM, David G, Dziezyc J, Humphrey JD (2007). Regional mechanical properties and stress analysis of the human anterior lens capsule. Vis. Res..

[26] Pedrigi RM, de Silva R, Bovens SM, Mehta VV, Petretto E, Krams R (2014). Thin-cap fibroatheroma rupture is associated with a fine interplay of shear and wall stress. Arterioscler. Thromb. Vasc. Biol..

[27] Pedrigi RM, Poulsen CB, Mehta VV, Ramsing Holm N, Pareek N, Post AL, Kilic ID, Banya WA, Dall’Ara G, Mattesini A, Bjorklund MM, Andersen NP, Grondal AK, Petretto E, Foin N, Davies JE, Di Mario C, Fog Bentzon J, Erik Botker H, Falk E, Krams R, de Silva R (2015). Inducing persistent flow disturbances accelerates atherogenesis and promotes thin cap fibroatheroma development in D374Y-PCSK9 hypercholesterolemic minipigs. Circulation.

[28] Raaz U, Kuhn H, Wirtz H, Hammerschmidt S (2009). Rapamycin reduces high-amplitude, mechanical stretch-induced apoptosis in pulmonary microvascular endothelial cells. Microvasc. Res..

[29] Sabbah HN, Khaja F, Hawkins ET, Brymer JF, McFarland TM, van der Bel-Kahn J, Doerger PT, Stein PD (1986). Relation of atherosclerosis to arterial wall shear in the left anterior descending coronary artery of man. Am. Heart J..

[30] Schneider CA, Rasband WS, Eliceiri KW (2012). NIH Image to ImageJ: 25 years of image analysis. Nat. Methods.

[31] Thacher T, Gambillara V, da Silva RF, Silacci P, Stergiopulos N (2010). Reduced cyclic stretch, endothelial dysfunction, and oxidative stress: an ex vivo model. Cardiovascular Pathology.

[32] Valentin A, Humphrey JD, Holzapfel GA (2011). A multi-layered computational model of coupled elastin degradation, vasoactive dysfunction, and collagenous stiffening in aortic aging. Ann. Biomed. Eng..

[33] Wang BW, Chang H, Lin S, Kuan P, Shyu KG (2003). Induction of matrix metalloproteinases-14 and -2 by cyclical mechanical stretch is mediated by tumor necrosis factor-alpha in cultured human umbilical vein endothelial cells. Cardiovasc. Res..

[34] Wang C, Baker BM, Chen CS, Schwartz MA (2013). Endothelial cell sensing of flow direction. Arterioscler. Thromb. Vasc. Biol..

[35] Wung BS, Cheng JJ, Chao YJ, Lin J, Shyy YJ, Wang DL (1996). Cyclical strain increases monocyte chemotactic protein-1 secretion in human endothelial cells. Am. J. Physiol..

[36] Zhang L, Liu Y, Zhang PF, Zhao YX, Ji XP, Lu XT, Chen WQ, Liu CX, Zhang C, Zhang Y (2010). Peak radial and circumferential strain measured by velocity vector imaging is a novel index for detecting vulnerable plaques in a rabbit model of atherosclerosis. Atherosclerosis.

